# Differential Localization of Structural and Non-Structural Proteins during the Bluetongue Virus Replication Cycle

**DOI:** 10.3390/v12030343

**Published:** 2020-03-20

**Authors:** Bjorn-Patrick Mohl, Adeline Kerviel, Thomas Labadie, Eiko Matsuo, Polly Roy

**Affiliations:** 1 Department of Infection Biology, London School of Hygiene and Tropical Medicine, London WC1E 7HT, UKAdeline.Kerviel@lshtm.ac.uk (A.K.); Thomas.Labadie@lshtm.ac.uk (T.L.); 2 Microbiology & Immunology, Division of Animal Science, Department of Bioresource Science, Graduate School of Agricultural Science, Kobe University, Kobe City 657-8501, Japan; eiko_matsuo@amethyst.kobe-u.ac.jp

**Keywords:** bluetongue virus, viral inclusion bodies, virus maturation, virus egress

## Abstract

Members of the *Reoviridae* family assemble virus factories within the cytoplasm of infected cells to replicate and assemble virus particles. Bluetongue virus (BTV) forms virus inclusion bodies (VIBs) that are aggregates of viral RNA, certain viral proteins, and host factors, and have been shown to be sites of the initial assembly of transcriptionally active virus-like particles. This study sought to characterize the formation, composition, and ultrastructure of VIBs, particularly in relation to virus replication. In this study we have utilized various microscopic techniques, including structured illumination microscopy, and virological assays to show for the first time that the outer capsid protein VP5, which is essential for virus maturation, is also associated with VIBs. The addition of VP5 to assembled virus cores exiting VIBs is required to arrest transcriptionally active core particles, facilitating virus maturation. Furthermore, we observed a time-dependent association of the glycosylated non-structural protein 3 (NS3) with VIBs, and report on the importance of the two polybasic motifs within NS3 that facilitate virus trafficking and egress from infected cells at the plasma membrane. Thus, the presence of VP5 and the dynamic nature of NS3 association with VIBs that we report here provide novel insight into these previously less well-characterized processes.

## 1. Introduction

Bluetongue virus (BTV) represents a robust model system for large, non-enveloped, double-stranded (ds) RNA viruses. BTV is transmitted by biting midges of the *Culicoides* species to ruminants and is endemic worldwide. BTV infection causes high morbidity and mortality in sheep and cattle, leading to significant economic consequences. The BTV genome consists of 10 dsRNA segments (S1–S10), which encode seven structural proteins (VP1–VP7) and four non-structural proteins (NS1–NS4) [1,2,3,4,5]. Among the structural proteins, VP1 (RNA-dependent RNA polymerase), VP4 (capping enzyme), and VP6 (helicase/RNA packaging) constitute the transcription complex. VP3 and VP7 form the inner capsid, which contains the transcription complexes and dsRNA segments, and is termed as a “core” particle. VP5 and VP2 comprise the outer capsid and their addition to the core particle is required for virus maturation.

VP5 is the membrane fusion and penetration protein of BTV that facilitates virus escape from late endosomes by sensing the low pH (∼5.5) and engaging its penetration activity [6]. VP5 consists of three domains: dagger (M1–S68), unfurling (K69–F354), and anchoring (I355–A526) domains [7]. Notably, the dagger domain, which is located at the N-terminus, is sequestered within the clefts between adjacent VP7 trimers that form the surface of the core and concealed from the external environment. The dagger domain interacts with adjacent VP7 molecules through charge complementarity and hydrophobic interactions. Each VP5 trimer bridges the channel formed by six surrounding VP7 trimers [7].

One characteristic of BTV infection is the formation of large globular structures called viral inclusion bodies (VIBs) in the infected cell cytoplasm, which lack a membrane [8,9]. The major component of the VIBs is the non-structural protein 2 (NS2). NS2 is able to bind positive-sense, single-stranded viral RNAs and the only BTV protein that is phosphorylated [10,11]. NS2 alone is sufficient for the formation of inclusion bodies in the cytoplasm [12]. NS2 functions as a concentrator or scaffold in the cytoplasm, recruiting the 10 viral positive-sense ssRNA transcripts, the transcription complex components, and inner capsid proteins into VIBs, to assemble core particles [12,13]. VIBs are the sites of virus replication and are believed to be the site of viral core assembly [14]. Furthermore, NS2 is indispensable for the assembly of the primary replicase complex that initiates secondary replication in the host cells [15].

While NS2 is indispensable for virus replication and core assembly, the non-stuctural protein 3 (NS3) protein is associated with intracellular virus trafficking [16]. During virus infection, NS3 is glycosylated and associates with intracellular membranes as well as the plasma membrane [9,17,18]. The carboxy-terminal of NS3 interacts with several cellular proteins, such as a subunit of the calpactin complex of the exocytic pathway, the cellular component p11 (S100A10), Tsg101, and the E3 ubiquitin-protein ligase NEDD4 family of proteins [16,19,20]. BTV has been found to require multi-vesicular-body (MVB) components and exocytic pathway proteins for infectious virus production [20]. NS3 also interacts with the outer capsid proteins VP5 and VP2, through its cytoplasmic N-terminal domain, hence hypothesized to be involved in virus maturation [9,16,21].

Although many studies have been undertaken on BTV replication at the molecular and structural level, the visualization of certain events during virus replication is still lacking, in particular, how virus core egress from VIBs and subsequently mature through the addition of the outer capsid. By using confocal and structured illumination microscopy techniques, we aimed to acquire a visual insight to elucidate the last steps of BTV replication, to facilitate a better understanding of virus assembly and intracellular viral trafficking. Here we report the association of the outer capsid protein VP5 with VIBs, the presence of which may facilitate the arrestation of transcriptionally active cores for exiting from VIBs [22]. We also report on our observations that during VIB formation, NS3 can be observed in VIBs during early stages of virus replication, but is re-localized to the periphery of the VIBs during later stages. Together, this sequencing of VP5 associating with transcriptionally active cores and the peripheral association of NS3 may act as a bridge, linking completion of virus maturation (via the final addition of VP2 to the core) and subsequent virus egress (via the exocytic pathway) at the plasma membrane.

## 2. Materials and Methods

### 2.1. Cell Lines and Viruses

BSR cells (BHK-21 subclone) (ATCC^®^ CCL10™) were maintained in Dulbecco modified Eagle medium (DMEM, Sigma-Aldrich, St. Louis, MI, USA) supplemented with 5% fetal bovine serum (FBS; Thermo Fisher Scientific, Waltham, MA, USA). The BTV serotype 1 (BTV-1) used in this study was recovered by reverse genetics. Reverse genetics was performed as previously described [15,23]. Briefly, at Day 1, 70%–80% confluent BSR monolayers were transfected with pCAG-VP1, pCAG-VP3, pCAG-VP4, pCAG-VP6, and pCAG-NS2 (120 ng each) using Endofectin (GeneCopoeia, Inc., Rockville, MD, USA), according to the manufacturer’s instructions, and incubated at 35 °C in 5% CO_2_ overnight. At Day 2, the cells were transfected with BTV-1 RNA transcripts, overlaid with 1% agarose and incubated for 3 days at 35 °C in 5% CO_2_. The recovered virus was plaque-purified, amplified in BSR cells, and harvested at a 100% cytopathic effect (CPE) for between 2 and 3 days. Viruses were titrated using a plaque assay. NS3 PBM mutant viruses (NS3_PBM1_ and NS3_PBM2_) were generated as previously described [9].

### 2.2. Antibodies and Reagents

Antibodies produced in the laboratory were used for detection of viral proteins NS2 (guinea pig, mouse or rabbit), NS3 (rabbit), VP5 (guinea pig), and VP6 (mouse or rabbit). Hoechst 33342 and Alexa 488-, 546- and 633-conjugated secondary antibodies were purchased from Life Technologies (Thermo Fisher Scientific, Waltham, MA, USA). Alkaline phosphatase-conjugated goat anti-mouse, anti-guinea pig, and anti-rabbit polyclonal antibodies, as well as paraformaldehyde (PFA), Fluoromount G, Bovine Serum Albumin (BSA), saponin, and Triton X-100 were purchased from Sigma-Aldrich (St. Louis, MI, USA). Mounting medium for structured illumination microscopy (SIM) sample preparation was purchased from Electron Microscopy Sciences (Hatfield, PA, USA).

### 2.3. Immunofluorescence Confocal and Structured Illumination Microscopy

**Figure 1:**
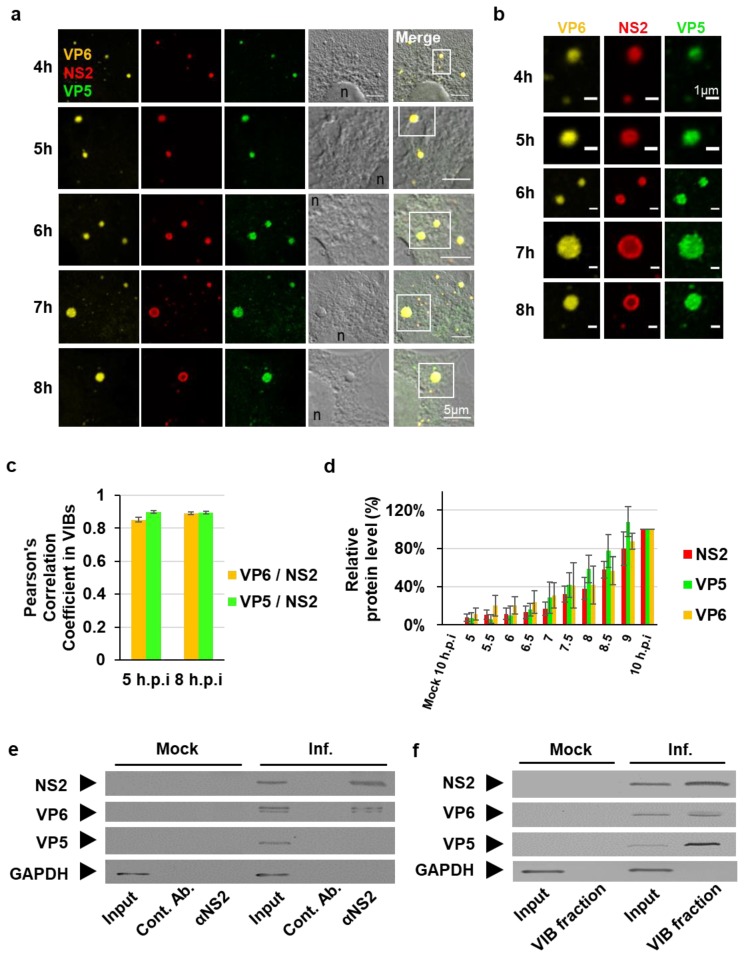
Both inner and outer viral capsid proteins can be found within VIBs. (**a**) Immunofluorescence analysis of the intracellular localization of the non-structural protein NS2 (red), the outer capsid protein VP5 (green), and the inner capsid protein VP6 (yellow) from 4h to 8 h.p.i., in cells infected at an MOI 5. Scale bar = 5 µm. n, nucleus. (**b**) Enlargement of section from (**a**) showing NS2, VP5, and VP6 individually. Scale bar = 1 µm. (**c**) Quantification of Pearson’s correlation coefficient between VP6 and VP5 with VIBs (NS2). The data are depicted as means with SE, *n* = 30. (**d**) Densitometry analysis of Western blot detection of the expression of the viral proteins NS2, VP5, VP6, and host cell GAPDH from 5 to 10 h.p.i. (Supplementary Figure S1). BSR cells were infected with BTV1 as above. The Western blots are expressed as a percentage, normalized to GAPDH. Error bars represent the SD values from three independent experiments. (**e**) Western blot detection of the viral proteins NS2, VP5, VP6, and host cell GAPDH at 8 h.p.i from Co-IPs using control antibody and GP-anti-NS2 antibody bound Protein A beads. BSR cells were infected with BTV1 as above. (**f**) Western blot detection of the viral proteins NS2, VP5, VP6, and host cell GAPDH at 8 h.p.i. from centrifugal enrichment of VIBs from cells treated as above.

**Figure 2:**
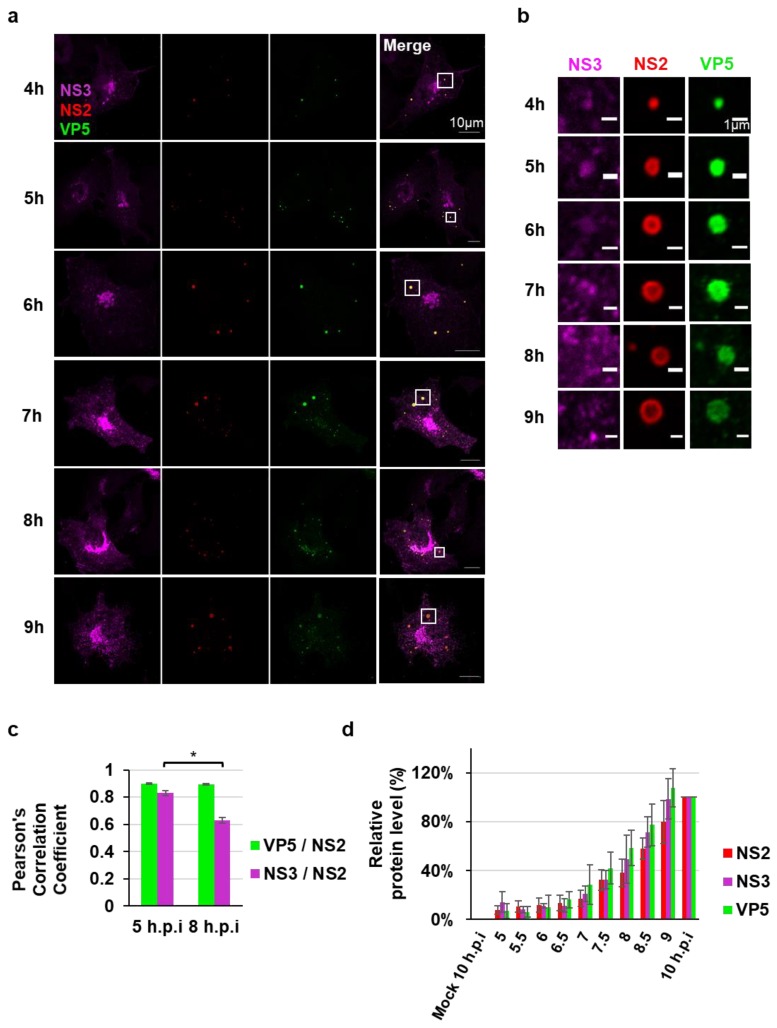
The dynamic nature of NS3 association with VIBs during the BTV replication cycle. Immunofluorescence analysis of the intracellular localization of the VIB-specific non-structural protein NS2 (red), the non-structural protein NS3 (magenta), and the outer capsid protein VP5 (green) from 4 h.p.i. to 9 h.p.i. Scale bar = 10 µm. (**b**) Enlargement of section from (**a**) showing NS2, VP5, and VP6 individually. Scale bar = 1 µm. (**c**) Quantification of Pearson’s correlation coefficient between VP5 and NS3 with VIBs (NS2). The data are depicted as means with SE, *n* = 30, * *p* < 0.01. (**d**) Densitometry analysis of the Western blots (Supplementary Figure S2) expressed as a percentage, normalized to GAPDH. Error bars represent the SD values from three independent experiments.

BSR cells (1.5 × 10^5^ cells) were grown on coverslips infected with BTV-1 at a Multiplicity of Infection (MOI) of 1 or 5. Cells inoculated with BTV were incubated at 4 °C for 1 h, and the inoculum then replaced with fresh cell culture media prior to incubation at 35 °C, thereby allowing us to synchronize virus entry and the subsequent virus time points. The unbound virus was removed, and infected cells were incubated at 35 °C. Cells were fixed at specified times post-infection with 4% (*w/v*) PFA, washed with PBS, and processed for immunofluorescence. Briefly, cells were either permeabilized with 0.5% Triton X-100 or 0.1% (*w/v*) saponin, blocked with 1% (*w/v*) BSA, and subsequently stained using specified primary and secondary antibodies diluted in 0.1% BSA. Images were acquired using a Zeiss LSM510 confocal microscope or a Zeiss LSM880 Airyscan confocal microscope (London School of Hygiene and Tropical Medicine) or a Zeiss LSM780 confocal microscope (National Institute of Health) using oil immersion 40×, 63×, or 100× objectives. Images were processed using ImageJ, Volocity, or Huygens Software. For SIM, samples were prepared as described above. Images were acquired using a Zeiss Elyra Ps1 microscope (Laboratory for Molecular Cell Biology, University College London, or Nihal Altan-Bonnet’s laboratory, NIH, Bethesda) equipped with an ×63 oil objective, and further processed and analyzed with the Zen software (Carl Zeiss Ltd., Hong Kong, China) and analyzed with ImageJ. PCC was calculated only for the VIBs in Figure 1c and Figure 2c. Using Volocity software, circular ROI were drawn around VIBs and cropped to selection. These images were then analyzed using the integral Volocity software measurement tools that calculated the PCC.

### 2.4. Western Blot

SDS–PAGE gels were transferred via a semi-dry blotter to PVDF transfer membranes and blocked for 1 h with TBS-T containing 10% (*w/v*) milk powder or 5% (*w/v*) BSA. Primary antibodies were added to membranes and incubated overnight at 4 °C, then alkaline phosphatase-conjugated secondary antibodies (IgG, Sigma-Aldrich, St. Louis, MI, USA) were added to the membrane for 1h at RT. Immunoblots were scanned with a gel doc system (Syngene G:BOX Chemi XRQ, Cambridge, UK) and protein levels were semi quantified using ImageJ.

### 2.5. Co-Immunoprecipitation Assay (Co-IP)

Protein A Sepharose beads (Sigma-Aldrich, St. Louis, MI, USA) were blocked with 1% BSA for 1 h at RT and resuspended at 100 mg/mL in lysis buffer (50 mM Tris-HCl (pH 7.5), 125 mM NaCl, 5% Glycerol, 0.2% NP-40, 1.5 mM MgCl_2_) supplemented with a cocktail of protease inhibitors (Halt™ Protease Inhibitor Cocktail (100X) Catalog #78430, Thermo Fisher Scientific, Waltham, MA, USA). A hundred microliters of Protein A Sepharose beads were incubated overnight at 4 °C with Guinee pig anti-NS2 antibodies and further washed three times with lysis buffer. BSR cells were infected with BTV-1 at an MOI of 5 for 1 h at 35 °C. At 8 h.p.i, cells were washed with cold PBS and resuspended in the lysis buffer. Following 30 min of incubation on ice, cell lysates were clarified and incubated overnight with the beads and gently rocked on ice. The beads were then centrifuged and washed four times with lysis buffer and resuspended in SDS containing loading buffer. Samples were analyzed by Western blotting.

### 2.6. Differential Velocity Centrifugation

VIB were purified from BTV1-infected BSR cells (MOI = 5) at 5 h.p.i and 8 h.p.i. Cells were washed with PBS before lysis with lysis buffer, as described for the co-immunoprecipitation assay. Cells were harvested and incubated on ice for 30 min in lysis buffer. Lysates were centrifuged at 800× *g* for 10 min to spin down nuclei and un-lysed cells. Supernatants were recovered and centrifuged at 6000× *g* for 20 min. The supernatant was removed without disturbing the white tight pellet (i.e., inclusion bodies) and upper jelly-like layer (membrane proteins). The pellet was resuspended in the Triton X-100/EDTA Solution (1% Triton X-100, 5 mM EDTA) and spun-down at 6000× *g* for 5 min. These washes and spins were repeated between 3 to 5 times to remove membrane contaminants until the jelly-like layer was no longer seen. Inclusion bodies were resuspended in SDS-loading buffer and analyzed by Western blotting.

### 2.7. Statistical Analyses and Software

Data were analyzed using ImageJ (NIH, Bethesda, MD, USA), Volocity (PerkinElmer, Waltham, MA, USA), or Huygens software (Scientific Volume Imaging, Hilversum, The Netherlands). T-tests were performed for statistical comparison.

## 3. Results

### 3.1. The Outer Capsid Protein VP5 Is Associated with VIBs

Following core release into the host cell cytosol, newly synthesized viral mRNA transcripts are released from the core to initiate the primary replication cycle [24,25]. Significant increases of viral mRNA are observed between 2 h post infection (h.p.i) and 8 h.p.i, and subsequent viral protein synthesis [24,26,27]. Here we sought to examine the distribution of viral proteins in the cytoplasm during this period. Following infection of BSR cells with BTV1 at an MOI of 5, viral protein expression was analyzed by immunofluorescence assays from 4 h.p.i to 10 h.p.i, using NS2 as the VIB marker, the inner capsid VP6, the helicase/RNA packaging protein, and the outer capsid protein VP5 (Figure 1a). Enlargements of sections from Figure 1a showing each protein individually indicate an overlap in signal of the respective channels (Figure 1b). The colocalization between VP6 and VP5 with NS2 was observed and quantified by using Pearson’s correlation coefficient (PCC) at 5 h.p.i and 8 h.p.i (Figure 1c). At 5 h.p.i and 8 h.p.i the mean PCC value of VP6 and NS2 were 0.85 (±0.01) and 0.89 (±0.01), respectively (Figure 1c, purple columns). Similarly, at 5 h.p.i and 8 h.p.i the mean PCC value of VP5 and NS2 were 0.9 (±0.01) and 0.89 (±0.01), respectively (Figure 1c, green columns). Since the PCC of VP5 was very similar to VP6, which is known to accumulate within VIBs and is a component of the replicase complex that is incorporated into virus cores [13,28], these results suggest VP5 is in the proximity of VIBs.

Complementary cell lysates of the immunofluorescence assay were analyzed by Western blots and viral protein expression was detected as early as 5.5 h.p.i for NS2, VP5, and VP6 (Figure S1). Densitometry quantification (Figure S1) confirmed that viral protein abundance progressively increased; however, no significant deviation of comparative protein abundance between the three proteins quantified could be observed at the individual time points (Figure 1d). This indicated uniform viral protein synthesis during the time course concluding at 10 h.p.i (Figure 1d).

To further investigate the association of VP5 with VIBs, we carried out Co-IP assays (Figure 1e) and differential velocity centrifugation of lysates of infected BSR cells at 8 h.p.i (Figure 1f). For Co-IP assays, cell lysates were incubated with protein A sepharose beads conjugated with a control serum or an anti-NS2 antibody. The protein complexes co-immunoprecipitated with the beads were eluted and analyzed by Western blots using antibodies against NS2, VP6, VP5, and GAPDH. Alongside NS2, a co-immuno-precipitating band was detected for VP6; however, no VP5 could be detected (Figure 1e, lane 6), suggesting that NS2 did not bind VP5. To further investigate this, we analyzed the contents of VIBs derived from differential velocity centrifugation (Figure 1f). Interestingly, purified VIBs contained VP6 and VP5 as well as NS2 (Figure 1f, Lane 4). Thus, we observed that VP5 colocalized with NS2 in IFA and also found it within VIBs; however, VP5 could not be observed in Co-IP samples. Cumulatively, these data suggest that VP5 may be considered as a separate marker for VIBs and as an indicator of viral assembly.

### 3.2. NS3 Association with VIBs During Viral Assembly

Since NS3 has been shown to be associated with VP5 previously during virus assembly and egress [21,29], we wanted to examine the localization of NS3 in relation to VIBs. NS3 is a membrane-associated glycosylated protein involved in virus trafficking and budding [9,16]. During the virus egress, NS3 interacts with VP5 to mediate intracellular trafficking to the plasma membrane [21]. To clarify further if NS3 is associated with VIBs together with VP5, the NS3 localization was examined from 4 h.p.i to 10 h.p.i. by confocal microscopy.

Upon co-staining NS2, NS3, and VP5, we observed all three proteins colocalizing at earlier time points; however, the NS3 association with VIBs appeared to evolve as the viral cycle progressed. The distribution of NS3 shifted from the inside of VIBs to their periphery between 4 h.p.i and 10 h.p.i (Figure 2a,b). The colocalization between VP5 and NS3 with NS2 was observed and quantified by using PCC at 5 h.p.i and 8 h.p.i (Figure 2c). At 5 h.p.i and 8 h.p.i the mean PCC value of VP5 and NS2 were 0.9 (±0.01) and 0.89 (±0.01), respectively. However, at 5 h.p.i and 8 h.p.i the mean PCC value of NS3 and NS2 were 0.83 (±0.01) and 0.62 (±0.02), respectively, representing a significant decrease.

To investigate further if changes in protein expression levels could be responsible for this observation, complementary cell lysates were assessed for viral protein expression. Viral proteins were detected as early as 5.5 h.p.i for NS2, VP5, and NS3 (Figure S2). Densitometry quantification of Figure S2 confirmed that viral protein abundance progressively increased; however, no significant deviation of comparative protein abundance between the three proteins quantified could be observed at the individual time points (Figure 2d). This indicated uniform viral protein synthesis during the time course concluding at 10 h.p.i (Figure 2d).

**Figure 3:**
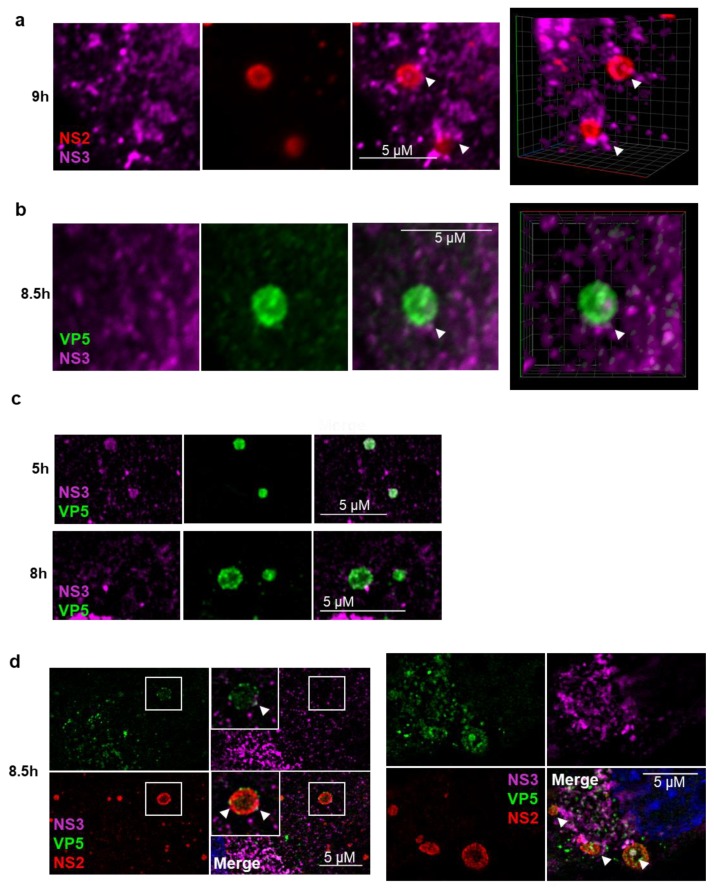
NS3 associates with the periphery of VIBs during the BTV replication cycle and outer capsid protein VP5. Immunofluorescence analysis of the localization of the VIB-specific non-structural protein 2 (NS2) and the non-structural protein 3 (NS3) and the outer capsid protein VP5. (**a**) VIB localization of NS2 (red) and NS3 (magenta) in BTV infected cells 9 h.p.i. (scale bar = 5 µm). Right panel: 3D projection of the z-stack. (**b**) VIB localization of VP5 (green) and NS3 (magenta) in BTV infected cells 8.5 h.p.i. (scale bar = 5 µm). Right panel: 3D projection of the z-stack. (**c**) VIB localization of the non-structural protein NS3 (magenta) and the outer capsid protein VP5 (red) at 5 h.p.i and 8 h.p.i. as observed by SIM (scale bar = 5 µm). (**d**) VIB localization of the non-structural protein NS3 (magenta), NS2 (red), and VP5 (green) at 8.5 h.p.i. as observed by SIM (scale bar = 5 µm). White arrows indicate peripheral association of NS3 with VIB.

To gain a more comprehensive understanding of the spatial distribution and arrangement of NS2, NS3, and VP5 in relation to one another, we generated 3D projections of z-stack images of BTV infected cells obtained by confocal microscopy. Beyond 8 h.p.i the 3D projections revealed NS3 on the periphery of NS2 labelled VIBs (Figure 3a and Video S1); similarly, NS3 could be visualized on the periphery of VP5 labelled VIBs (Figure 3b and Video S2). BTV infected cells co-stained for NS3 and VP5 at 5 h.p.i and 8 h.p.i also revealed a similar shift of NS3 away from VIB structures labelled with VP5 as seen by SIM (Figure 3c). Furthermore, labelling BTV infected cells with NS2, NS3, and VP5 showed NS3 at the periphery of NS2/VP5-labelled VIBs with some colocalization of NS3 and VP5 at the periphery of the VIBs (Figure 3d). All together, these observations describe a shift of NS3 to the periphery of the VIBs, possibly localizing to endoplasmic reticulum and Golgi apparatus membranes [9]. Further, the colocalization of NS3 and VP5 at the periphery of the VIBs may indicate the bridging site where NS3 captures partially mature particles facilitating virus maturation and trafficking out of the cell.

**Figure 4:**
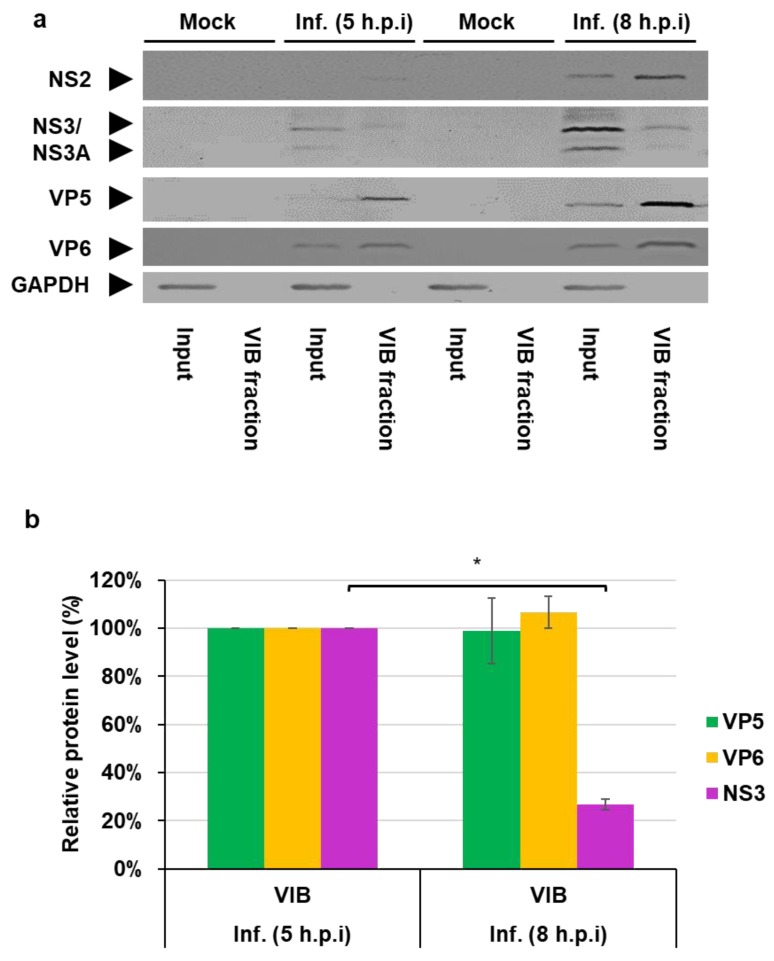
NS3 association with VIBs decreases during the BTV replication cycle. Analysis of cell lysates of mock and infected BSR cells. (**a**) Western blot detection of the viral proteins NS2, VP5, VP6, NS3, and host cell GAPDH at 5 and 8 h.p.i from differential velocity centrifugation for VIBS from cells infected at an MOI of 5. (**b**) Densitometry analysis of the Western blots (a) expressed as a percentage, normalized to NS2. Error bars represent the SD values from three independent experiments. * *p* < 0.01.

To further elucidate the shift of NS3 away from VIBs and confirm our immunofluorescence microscopy data, we analyzed the constituents of VIBs derived from differential velocity centrifugation at 5 h.p.i and 8 h.p.i. Western blot analysis of VIBs indicated the presence of NS2, VP6, VP5, and NS3 (Figure 4a). Host cell GAPDH was used as a cellular marker protein. We then analyzed the ratio of NS3, VP5, or VP6 with NS2 that was used as a marker, indicating the VIBs concentration. Relative to NS2, NS3 protein levels decreased by 73% ± 2% in the VIB enriched fraction at 8h.p.i. compared with 5 h.p.i; no such significant change could be observed for VP5 or VP6 (Figure 4a,b). These data are consistent with our immunofluorescence data showing a decrease of NS3 colocalizing with NS2 of VIBs (Figure 2c). However, no such shift was observed for VP5 or VP6 (Figure 4a,b).

### 3.3. NS3 Trafficking Mutants Affect VP5 Export to the Plasma Membrane

**Figure 5:**
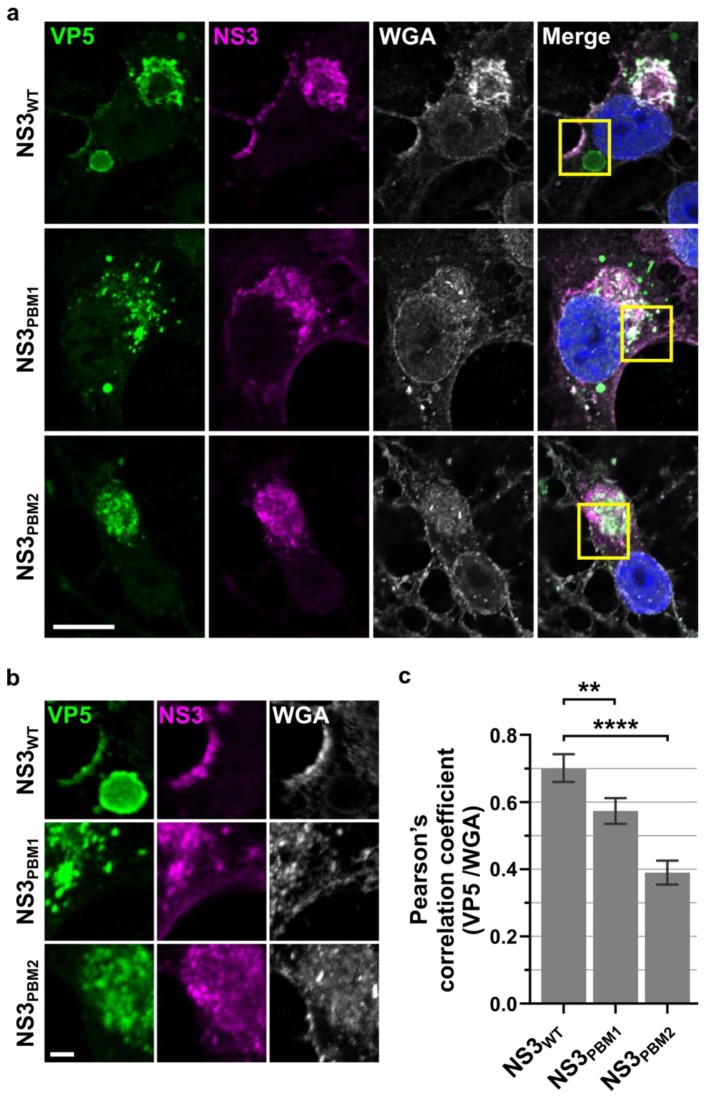
NS3 trafficking mutants affect VP5 export to the plasma membrane. (**a**) Confocal microscopy images of VP5 (green) and NS3 (purple) in BSR cells infected by either WT BTV (top row), BTV NS3PBM1 (middle row), and BTV NS3PBM2 (bottom row) viruses 24 h.p.i, and labelled with the membrane fluorescent marker Wheat germ agglutinin (WGA, white). The nucleus is shown in blue, and the scale bar represents 10 μm (white line, bottom left corner). The squares represent the region of interest magnified and shown in (**b**), where the scale bar represents 2 µm (white line). The colocalization between WGA and VP5 in whole cells was quantified for each virus using (**c**) the Pearson’s correlation coefficient. The data are depicted as means and SD; *n* = 30; ** *p* < 0.01; **** *p* < 0.0001).

Following the redistribution of NS3 to the periphery of the VIBs and its colocalization with VP5 at these sites of contact, we next examined the trafficking of VP5 to the plasma membrane. Recently we have reported the importance of two polybasic motif (PBM) located within NS3 that influence its cellular trafficking [9]. Since mutation at the PBM motifs of NS3 perturbed its cellular trafficking and prevented trafficking to the plasma membrane, we investigated whether these mutations would perturb the localization of VP5. BSR cells were infected with the WT virus and the two PBM mutant viruses, in which the basic amino acids had been replaced by alanine. VP5 was clearly visible at the plasma membrane in association with NS3, as well as in the cytoplasm within or near the VIBs in the WT-infected BSR cells (Figure 5a,b). In cells infected with mutant viruses, VP5 was no longer associated with NS3 at the plasma membrane, and was distributed within the cytoplasm. Further, cells infected with NS3_PBM2_, the second site mutants, showed a much more severe effect on VP5 distribution in the cytoplasm compared to the cells infected with NS3_PBM1_ (Figure 5A,B). A quantitative analysis with the PCC (Figure 5C) revealed that NS3 and VP5 colocalization was slightly higher in the presence of WT NS3 (0.26 ± 0.008) than in the presence of NS3_PBM1_ (0.23 ± 0.006), and was significantly lower in the presence of NS3_PBM2_ (0.19 ± 0.004). These data further substantiate the bridging function of NS3 for the trafficking of a virus to the plasma membrane, as a perturbance of the PBMs, PBM1 as an ER retention signal and PBM2 mediating NS3 export from the Golgi apparatus to the plasma membrane [9], interfere with VP5 trafficking.

## 4. Discussion

While it has been established that the virus factories or VIBs utilized by members of the *Reoviridae* family are responsible for replication and the assembly of virus particles [30,31,32], crucial stages of these processes remain ill-defined. This includes the egress of virus cores from VIBs, and in the context of BTV, their subsequent associating with the outer capsid proteins VP5 and VP2 to generate mature infectious virus particles that are ready for egress from the cell. This report aims to further illuminate this latter stage of the virus lifecycle.

Following virus uncoating of the outer capsid proteins VP2 and VP5, and the release of cores into the cytosol, the latter become transcriptionally active. It has been shown that the detachment of the outer proteins during virus entry induces both global movements of the capsid shell and local conformational changes of an interacting VP3. This leads to structural changes of the RNA-dependent RNA-polymerase VP1 and its associated VP3 capsid proteins, priming the VP1 within the capsid for transcription to be engaged [33]. Furthermore, it has been reported for the rotavirus capsid, which is similar to the BTV capsid, that once transcription is engaged, 12 RNA exit channels, each placed at the fivefold vertices of the icosahedral capsid, simultaneously extrude mRNA [34,35].

In this context, we hypothesize a mechanistic purpose and rationale for the presence of VP5 at the VIBs, namely the occlusion of channels in newly assembled VP3/VP7 cores, to deprive them of access to transcription substrates (ATP and nucleotides). Sequentially, VP3 would recruit VP7 to the VIBs to assemble cores [12], before the addition of VP5. This would prevent viral mRNA transcription and transcript extrusion via these channels, as it has been found that each VP5 trimer bridges the channel formed by six surrounding VP7 trimers [7]. This could lock the virus into a primed state to facilitate the assembly of infectious virus for egress.

Following the association of the outer capsid protein VP5 with the cores, the presence of NS3 at the periphery of VIBs, as reported here, would be of significance. NS3 localizes to the endoplasmic reticulum, trafficking through the Golgi apparatus before reaching the plasma membrane [16,20]. Uniquely, NS3 interacts with cellular exocytosis factors [16,19,20,29,36] via its C-terminal domain and outer capsid proteins VP5 and VP2 via its N-terminal domain [16,21,37], but not NS2 (unpublished observation, Roy). As was shown here using SIM and confocal fluorescence microscopy techniques, at 7–8 h.p.i, VP5 was found around and inside VIBs and that NS3 localized mainly at the periphery of the VIBs, partially enveloping these. When NS3 is peripheral to VIBs, it could co-localize with VP5, indicating potential interphase sites of viral assembly/exit of “core” plus VP5 particles (immature particles) from the VIBs. Thus, viruses could assemble in or at the border of the VIBs and associate with NS3. Based on the viral architecture, VP5 is assembled on the surface of the core particles prior to VP2 assembly [7]. Consequently, NS3 could function as a bridge between the immature particles and VP2 and bring the newly formed particles to the plasma membrane by utilizing the host cell membrane trafficking flux of the exocytosis machinery. We found that this capacity is dependent upon the PBMs present within NS3 to facilitate trafficking to the plasma membrane, sequentially via NS3_PBM1_, which functions as a ER retention signal, and subsequently NS3_PBM2_, which facilitates Golgi apparatus export of NS3 to the plasma membrane [9].

Having shown the presence of VP5 at VIBs, juxtaposed to the observation that NS2 does not appear to bind and recruit VP5 to the VIBs, as it does in the inner capsid component VP6, a further component, such as a host factor, may be responsible. Future research will be needed to further refine our understanding of the proximity of the ER/Golgi network, the NS3 associated with it, and the VP5 around the VIBs.

## 5. Conclusions

This report contributes to the understanding of the sequential orchestration of virus assembly and maturation. VIBs act as a scaffold for the VP5/NS3 interaction. BTV may require the spatial proximity of outer capsid protein VP5 at VIBs, alongside other viral proteins, to stabilize and lock newly assembled core particles. Subsequently, the proximity of NS3 at the periphery of VIBs and the shown overlap of VP5 and NS3 at these sites may facilitate a conveyer belt-like organization; VP5 connecting to NS3 and drawing out cores from the site of assembly. Following this, the particles mature with the subsequent addition of VP2, before utilizing the exocytosis machinery to reach the plasma membrane and egressing from the cell. In summary, this allows us to propose an updated model of BTV virus assembly.

## Supplementary Materials

The following are available online at https://www.mdpi.com/1999-4915/12/3/343/s1, Figure S1: Western Blot detection of the expression of the viral proteins NS2, VP5, VP6 and host cell GAPDH, Figure S2: Western Blot detection of the expression of the viral proteins NS2, VP5, NS3 and host cell GAPDH. Video S1: 3D projection of the VIB localization of NS2 (red) and NS3 (magenta) in BTV infected cells 9 h.p.i. Video S2: 3D projection of the VIB localization of VP5 (green) and NS3 (magenta) in BTV infected cells 8.5 h.p.i.
