# Supplementary material for: Differential Localization of Structural and Non-Structural Proteins during the Bluetongue Virus Replication Cycle
Source: Viruses. 2020 Mar 20;12(3):343. doi: 10.3390/v12030343 (PMC7150864; doi:10.3390/v12030343)
Supplement: Supplementary file 1 [file viruses-12-00343-s001.zip › Supplementary-fig-S2.pdf]

Figure. S2

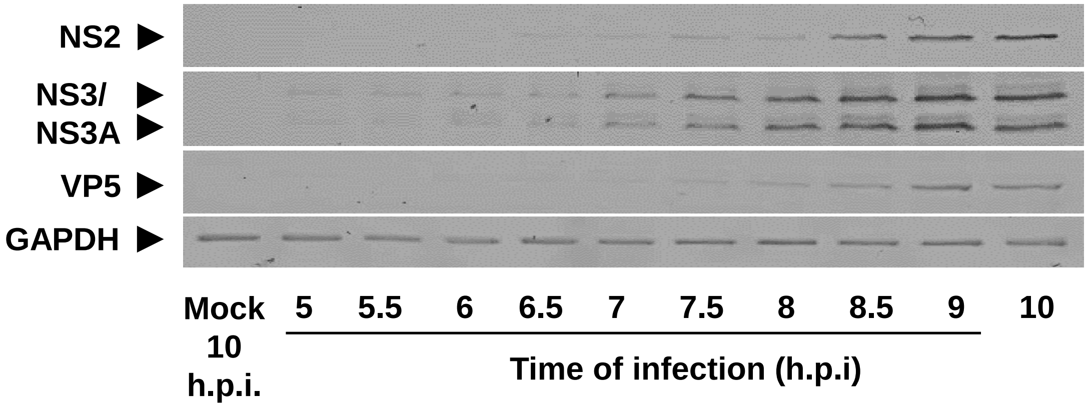

Figure. S2 Western Blot detection of the expression of the viral proteins NS2, VP5, NS3 and host cell GAPDH from 5 to 10 hours post infection (h.p.i). BSR cells were infected with BTV1 at MOI 5.
